# Event-based real-life outcomes of patients with non-neuronopathic Gaucher disease receiving ert

**DOI:** 10.1186/s13023-025-03690-8

**Published:** 2025-06-12

**Authors:** Ayşe Akyüz, Aslı İnci, İlyas Okur, Leyla Tümer, Fatih Süheyl Ezgü

**Affiliations:** 1 Faculty Of Medicine, Department of Pediatrics, Department of Inborn Metabolic Diseases, Gazı University, Eminiyet Mahallesi, Mevlana Bulvarı no:29, Yenimahalle, Ankara, 06560 Turkey; 2 Department of Inborn Metabolic Diseases , Ankara Training and Research Hospital, Talatpaşa Bulvarı no:128, Zekai Tahir Burak Ek Binası, Altındağ, Ankara, 06230 Turkey

**Keywords:** Gaucher, Lysosomal storage diseases, Enzyme replacement therapy

## Abstract

**Background:**

Gaucher Disease (GD) is a lysosomal storage disorder. Mutations in the GBA1 gene cause glucocerebrosidase enzyme deficiency that leads to the accumulation of its substrates. Enzyme replacement therapy emerged as a natural history-changing treatment. Up to now, mainly measurable treatment targets have been investigated for patients. In this study, the number of real-life based and GD related events before and after ERT were evaluated for the first time to assess the impact of ERT on GD patients in real-life settings. The events investigated consist of hematopoietic, musculoskeletal, gastrointestinal, neurologic, respiratory system and growth and puberty-related events, as well as events related to routine daily activities, and malignancy.

**Results:**

A total of 29 events were reported from 5 different group of events between − 12 to -6 months and 16 events from − 6 months to baseline in all patients. After the initiation of ERT, the number of new events decreased to 1 in 6–12 months. At the end of the follow-up period, between 30 and 36 months, only 1 new event was recorded, the minimum event number overall. For all groups of events, there was a trend to decrease of the events for overall follow-up period.

**Conclusion:**

In conclusion, this is the first study that evaluate event-based outcomes in GD patients receiving ERT and show the real-life data by evaluating not only laboratory parameters but also clinical consequences of the treatment, providing practical clinical follow up.

**Supplementary Information:**

The online version contains supplementary material available at 10.1186/s13023-025-03690-8.

## Background

Gaucher Disease (GD) (OMIM #230800) is a lysosomal storage disorder and one of the most common sphingolipidosis. Mutations in *GBA* gene cause glucocerebrosidase (Gcase) enzyme deficiency that leads accumulation of its substrates, glucosylceramide (GlcCer) and glucosylspingosine (GlcSph) in macrophages, named Gaucher cells. Gaucher cells are responsible for clinical symptoms by infiltrating various organs, especially bone marrow, spleen and liver [1, 2]. Main clinical presentations of GD are cytopenia, hepatosplenomegaly, bone involvement and, in neuronopathic forms (GD type 2 and 3), neurological impairment. Onset of the disease manifestations vary and can be considered at any age, but the median age of diagnosis is reported from 10 to 20 years old [3, 4]. Besides; abdominal pain, painful bone crises, bleeding, growth retardation, delayed puberty, pulmonary involvement, fatigue are other manifestations that severely affect daily life of Gaucher patients [5]. Currently there are two main specific therapies for GD: enzyme replacement therapies (ERT) and substrate reduction therapies (SRT). Recombinant enzymes have been developed (imiglucerase, velaglucerase, taliglucerase and they have been using since 1990s as ERT. It has been shown that ERT decreases spleen and liver volumes, improves hemoglobin and platelet count, reduces bone pain or crises [6, 7]. However, little is known about the effects of these measurable targets on real-life and it is not difficult to guess that GD has significant other real-life effects, which cause a significant disease burden and also reduce quality of life. This study aims to investigate the number of real-life based and GD related events before and after ERT beyond previously investigated and measurable treatment targets.

## Material and methods

This study was conducted in Gazi University Faculty of Medicine at the Department of Inborn Errors of Metabolism, in Ankara, Turkey. It was performed in line with the principles of the Declaration of Helsinki. Ethics committee approval was obtained from Gazi University, decision number is 04. Then, the data were collected retrospectively from patient’s medical records between 1997 and 2022. Patients over 2 years of age who were followed up at Gazi University Medical Faculty Hospital and whose clinical data were available for 1 year before and 3 years after the initiation of treatment were included in the study. In this retrospective study, all clinical data were obtained from the registered data of patients in the department.

All patients had enzymatically and/or genetically confirmed diagnosis of GD. Event-based and clinical data were obtained from patient’s routine follow up visits or from medical records or from phone or video calls. All clinical data and events were collected from patients’ medical records obtained with investigation upon GD symptoms during routine follow up. Routine follow up data included physical examination, anthropometrics (length/height, weight, and pubertal status), blood count (hemoglobin and platelet levels), liver and spleen volumes (by examination and by ultrasonographic measure), bone mineral density measures, concomitant medications or admission to any other department, information about bone pain, abdominal pain, ability of performing daily activities (school or work continuity), bleeding problems.

Real-life parameters were selected as important events which could result in significant morbidity, disability and reduction in quality of life but also which have the potential to be affected by treatment. In addition to the traditional therapeutic goals of ERT such as bleeding tendency, hepatosplenomegaly and skeletal pathologies current long-term goals for management of GD are defined within the event groups by focusing the morbidity and quality of life of patients [8–10]. The current long-term management and follow up parameters of GD including pulmonary complications, monitoring of growth, malignancy, neurological manifestations, assessment of quality of life caused by the fatigue and/or participation in daily activities are considered different groups of events and standardized.

These consist of hematological events (long-lasting and/or frequent bleeding episodes including abnormal uterine bleeding, hematuria, epistaxis (at least once a week), bleeding gums, erythrocyte or thrombocyte suspension transfusion, significant fatigue interfering with daily activities), skeletal events (bone fracture, avascular necrosis, need to use walking aids or wheelchair, bone pain interfering with routine daily activities, need to use continuous analgesic due to bone pain, bone pain causing depression), gastroenterological events (feeding difficulty and/or abdominal distention resulting from organomegaly interrupting physical activity, poor appetite or vomiting interrupting weight gain), events related to growth and puberty (growth retardation (the height below − 2SD and delayed puberty that needs to be followed up in the endocrinology unit), respiratory events (decrease in walking distance and/or impaired effort capacity related to respiratory dysfunction, need of ventilatory support (oxygen, mechanical ventilation etc.), need to use medication for poor respiratory function (inhaler steroids and/or salbutamol etc.), sleep problems), neurological events related to Parkinson disease such as tremor, bradykinesia, speech changes, impaired balance, rigid muscles, events related to quality of life (Inability to self-care, missing school or workdays, any psychiatric abnormality related to Gaucher Disease), malignancy events and Gaucheromas (Gaucheromas, hematological malignancies).

Events were collected starting from 1 year before and up to 3 years of ERT and all events were collected every 6 months periods. The patients have received ERT with a dose of 60 U/Kg/ every 2 weeks. Events that persisted or remained stable were counted only once at the occurrence of event or restarted after a six months period.

Statistical analysis was conducted with event numbers of a period of 12 months. Statistical analysis was performed using SPSS version 25 (SPSS Inc.). Wilcoxon test was used to ascertain the differences of number of events before and after initiation of ERT with a significance value set at *p* < 0,05.Odds Ratios for hematological events were calculated by using chi-square. Skeletal, gastroenterological, growth and puberty related and quality of life related events were calculated by using Fisher exact test due to because of the minimum expected count below 5.

## Results

### Demographics

**Fig. 1:**
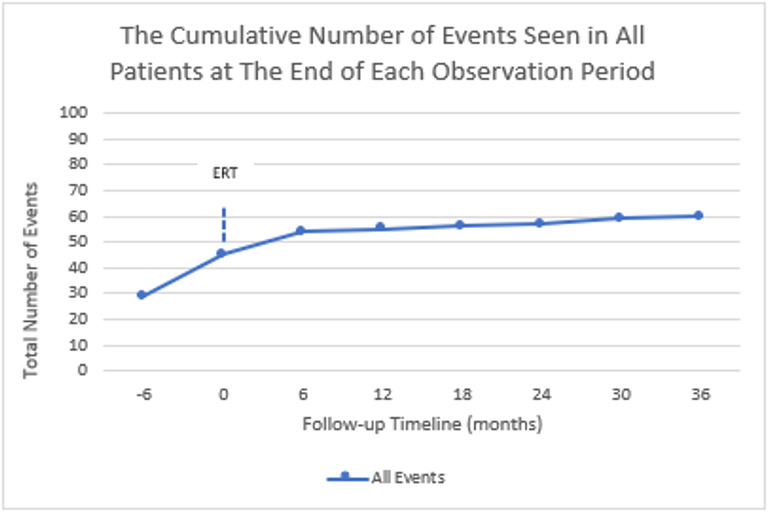
The cumulative number of events seen in all patients

A total of 15 non-neuronopathic patients, nine females and six males, were treated by ERT. Age at the initiation of ERT ranged from 2,5 to 52 years, with a mean age of 17,7 years. Five patients were below 10 years at initiation of treatment. All patients were diagnosed with non-neuronopathic GD as having at least one p.N409S mutation except one patient (patient 14) with p.R502C/F167V mutations who hasn’t any neurological involvement. Eight patients had organomegaly, five patients had cytopenia, one patient had bleeding abnormalities, and one patient had bone pain as the first symptom enabling the diagnosis of GD. All patients were started on ERT soon after the diagnosis except two patients (patients 5 and 6 who are twins), who were treated 11 years later. All patients were treated with imiglucerase and three patients switched to taliglucerase. Enzyme activity assay results and *GBA1* mutation analysis are listed below (Table 1). Duration of follow-up varied between 2 and 18 years with a mean 9,5 years. Event numbers and cumulative event numbers were listed in Fig. 1.

**Table 1:** Demographic, molecular and treatment data of gaucher patients

GD type I Cases	Sex	Main Symptom at diagnosis	Age at Diagnosis (years)	Glucocerebrosidase Enzyme activity (nmol/h/mg protein) (reference values)	GBA1 mutations	Age at initiation of Treatment (years)	Recombinant Enzyme Used
Patient 1	F	cytopenia	28	0,53 (> 7)	N409S/N409S	29	imiglucerase
Patient 2	M	bone pain	14	2,36 (30–80)	N409S/N409S<	14	imiglucerase
Patient 3	F	HSM	5	3,9 (30–80)	N409S/?	6	imiglucerase
Patient 4	M	HSM	51	0,24 (> 7)	N409S/N409S	52	imiglucerase
Patient 5	F	SM	9	0,11 (> 7)	N409S/N409S	22	Imiglucerase/taliglucerase
Patient 6	F	SM	9	0,11 (> 7)	N409S/N409S	22	Imiglucerase/taliglucerase
Patient 7	F	SM	13	1,83 (> 7)	N409S/N409S	13	Imiglucerase
Patient 8	M	HSM	16	10 (30–80)	N409S/?	17	imiglucerase
Patient 9	F	cytopenia	26	1,59 (> 7)	N409S/N409S	28	imiglucerase
Patient 10	M	cytopenia	4	NA	N409S/R464H	4	imiglucerase
Patient 11	M	cytopenia	8	0,73 (> 7)	N409S/N409S	8	imiglucerase
Patient 12	M	bleeding	2,5	0,73 (> 7)	N409S/L483P	2,5	imiglucerase
Patient 13	F	HSM	6	1,8 (> 7)	N409S/N409S	6	imiglucerase
Patient 14	F	cytopenia	9	1,21 (> 7)	R502C/F167V	29	Imiglucerase/taliglucerase
Patient 15	F	HSM	13	0,04 (> 7)	N409S/?	13	imiglucerase

*F: female, M: male, SM: splenomegaly, HSM: hepatosplenomegaly, NA: not available

None of the patients had splenectomy

### Hematological events

Before the initiation of ERT seven patients has epistaxis with minor trauma (patients 4,5,6,8,11,14,15) and three of them had frequent epistaxis especially during summer (patient 5,6,14). Patient 1 had been already splenectomized one year before the diagnosis of GD. Patient 13 had bleeding during first trimester of pregnancy putting the baby at risk, so she was restarted on ERT. On her follow up no complications or bleeding has occurred after initiation of ERT. Patient 9 had massive bleeding due to abnormal platelet functions while giving birth and needed six erythrocyte suspensions transfusion. Abnormal menstrual bleeding had still continued to be a problem despite ERT for patient 9 without any gynecological problem. Four patients needed at least one transfusion as erythrocyte suspension (patient 8,9) or thrombocyte suspension due to bleeding related to GD (patient 1,15). Number of hematological events significantly decrease in 12 months after initiation of ERT and no events were recorded afterward (Table 2).

**Table 2:** Assessment of number of different group of events comparing one year period before and after initiation of ERT

Events		Total Number of Events	Median (min-max)	*p*
Hematological Events	-12 months to baseline	21	2 (0–3)	
	Baseline to 12 months	4	0 (0–1)	0,004*
Skeletal Events	-12 months to baseline	7	0 (0–3)	
	Baseline to 12 months	2	0 (0–1)	0,102
Gastroenterological Events	-12 months to baseline	4	0 (0–2)	
	Baseline to 12 months	2	0 (0–1)	0,157
Respiratory Events	-12 months to baseline	NA		
	Baseline to 12 months	NA		
Events Related to growth and puberty	-12 months to baseline	7	0 (0–1)	
	Baseline to 12 months	0	0 (0–0)	0,008*
Neurological Events Related to Parkinson Disease	-12 months to baseline	NA		
	Baseline to 12 months	NA		
Tumoral Events	-12 months to baseline	NA		
	Baseline to 12 months	NA		
Events Related to Quality of Life	-12 months to baseline	6	0,4 ± 0,82	
	Baseline to 12 months	2	0,13 ± 0,35	0,102

### Gastroenterological events

Two patients (patients 8,11) had suffered from abdominal distention leading feeding difficulties caused by massive organomegaly. Although one patient’s (patients 8) massive organomegaly persisted but their feeding difficulties resolved after initiation of ERT.

### Skeletal events

Bone pain requiring having analgesic or blocking daily activities was seen in three patients (P2,11,14). Patient 11 could not participate monthly sports in his school during one year before ERT. He started playing sports more than 6 months after starting ERT.

All pain complaints decreased and patients no longer needed having medications except one, the patient 14 and continued their daily activities after ERT. Patient 14 suffered from vertebral fracture six months before initiation of ERT. This patient had osteoporosis and was using analgesics after several years of ERT. Only patient 2 had severe scoliosis requiring physiotherapy, probably not related to GD.

### Respiratory events

None of patients had GD related respiratory system event. Several years later of follow up period, patients 4 started using inhaler therapy because of decreased functional vital capacity with interstitial lung disease that was linked to GD by the pulmonologist following-up the patient.

### Events related to growth and puberty

**Fig. 2:**
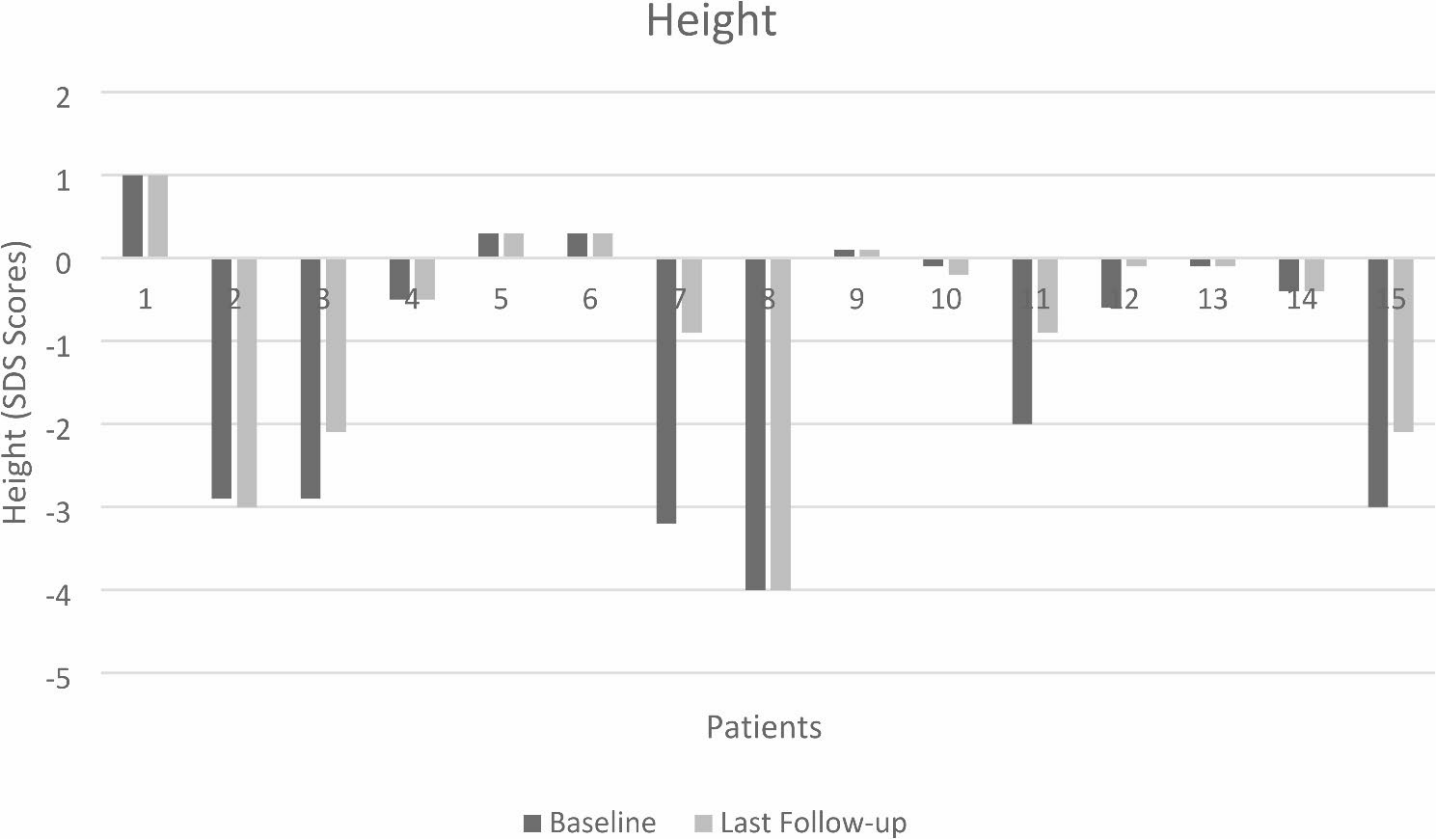
The height SDS scores of patients

The height was below − 2 standard deviations in six patients before treatment (Patients 2,3,7,8,11,15) necessitating pediatric endocrinology consultation. Two patients (patient 7,11) had acceleration in growth after ERT and reached normal height at the end of three years and discontinued pediatric endocrinology follow-ups (Fig. 2). These were the two of the five patients who started treatment in the prepubertal period. Other patients could not achieve normal growth rate. Since, no new event related to growth and puberty was recorded during the follow up that reflect a significant decrease of number of events (Table 2). Three patients had story of delayed puberty but they had diagnosed with GD after several years of this complaint.

### Events related to maintenance of daily activities

Three of the patients (Patient 2,9,14) were unable to go to work because of fatigue, pain, or abdominal distention. Despite ERT, two patients’ conditions remained stable and disability to go to work did not improve.

### Parkinson disease

None of the patients had Parkinson symptoms or any other neurological impairment.

### Events related to malignancy or Gaucheromas

Only one patient (patient 4) had splenic Gaucheromas. None of the patients had any malignancy.

### Event-based outcomes in general

A total of 29 events were reported from 5 different group of events between − 12 to -6 months and 16 events from − 6 months to baseline in all patients. It was noticed that after the initiation of ERT, the number of new events significantly decreased to 1 in 6–12 months (Table 3). At the end of the follow-up period, between 30 and 36 months, only 1 new event was recorded, the minimum event number overall. For all groups, there was a trend to decrease or stabilization of the events. All event numbers were listed in Table 4.

**Table 3:** Assessment of total number of events comparing one year period before and after initiation of ERT

	Total Number of Events	Median (min-max)	*p*
-12 months to baseline	45	2 (0–7)	
Baseline to 12 months	10	1 (0–2)	0.001 *
12 to 24 months	2	0 (0–1)	0.001*
24 to 36 months	3	0 (0–2)	0.001*

**Table 4:** Number of events seen in all patients

Events	-12 to -6 months	-6 to baseline	Baseline to 6 months	6 to 12 months	12 to 18 months	18 to 24 months	24 to 30 months	30 to 36 months
Hematological Events	14	7 (21)	4 (25)	0 (25)	0 (25)	0 (25)	0 (25)	0 (25)
Skeletal Events	3	4 (7)	2 (9)	0 (9)	0 (9)	0 (9)	1 (10)	1 (11)
Gastroenterological Events	2	2 (4)	2 (6)	0 (6)	0 (6)	0 (6)	0 (6)	0 (6)
Respiratory Events	0	0 (0)	0 (0)	0 (0)	0 (0)	0 (0)	0 (0)	0 (0)
Events Related to growth and puberty	7	0 (7)	0 (7)	0 (7)	0 (7)	0 (7)	0 (7)	0 (7)
Neurological Events Related to Parkinson disease	0	0 (0)	0 (0)	0 (0)	0 (0)	0 (0)	0 (0)	0 (0)
Tumoral Events	0 (0)	0 (0)	0 (0)	0 (0)	0 (0)	0 (0)	1 (1)	0 (1)
Events Related to Quality of Life	3	3 (6)	1 (7)	1 (8)	1 (9)	1 (10)	0 (10)	0 (10)
Total Events	29	16 (45)	9 (54)	1 (55)	1 (56)	1 (57)	2 (59)	1 (60)

*Number of events (number of cumulative events)

The most common real-life interfering events before ERT were related to the hematopoietic system. Hematopoietic system and growth and puberty are the group of events that the most striking significant decrease was seen However, odds ratios for skeletal events and events related to quality of life were consistent with treatment effect (Table 5).

**Table 5:** Assessment of number of patients experienced different group of events comparing one year period before and after initiation of ERT

Events	Patients experienced Events in -12 months to baseline	Patients experienced Events in Baseline to 12 months	Odds Ratio (95% CI)	*p*
Hematological Events	10	4	0.18 (0,03 − 0,87)	0,028*
Skeletal Events	3	2	0,66 (0,09 − 4,7)	1,0
Gastroenterological Events	2	2	1 (0,12 − 8,2)	1,0
Respiratory Events	NA	NA		
Events Related to growth and puberty	7	0	NA	0,006*
Neurological Events Related to Parkinson Disease	NA	NA		
Tumoral Events	NA	NA		
Events Related to Quality of Life	3	2	0,61 (0,08 − 4,3)	1,0

Hematological events were calculated by using chi-square. Skeletal, gastroenterological, growth and puberty related and quality of life related events were calculated by using Fisher exact test due to because of the minimum expected count below 5

**Fig. 3:**
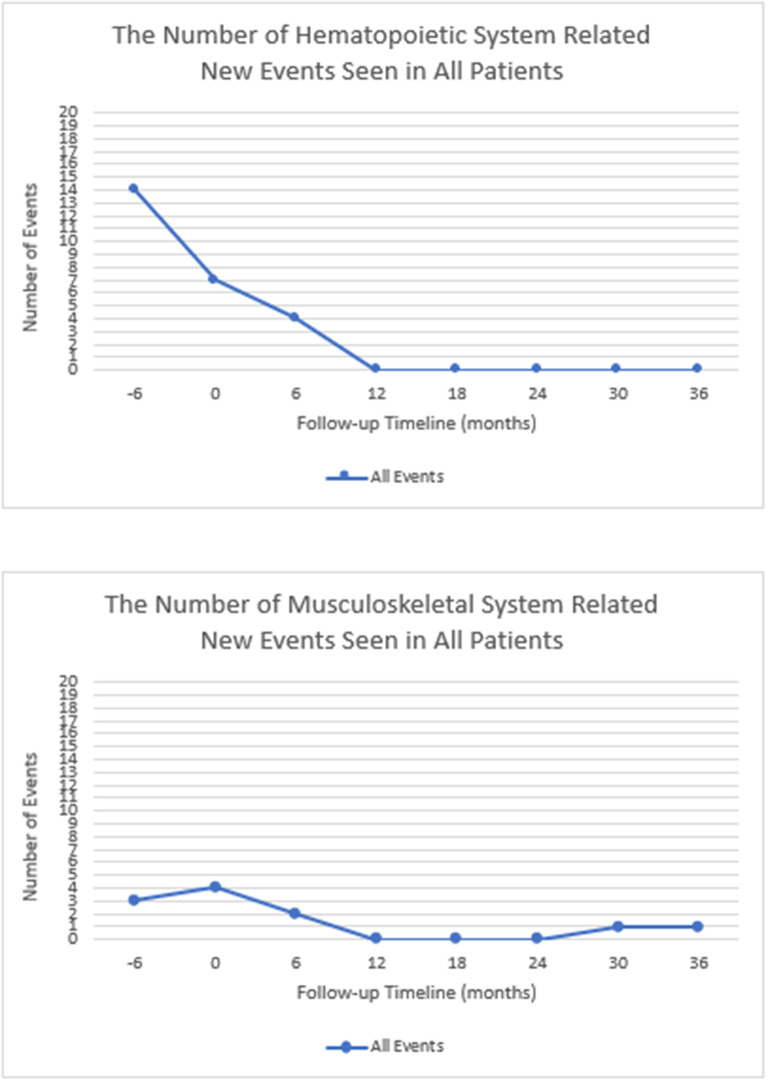
The number of hematopoietic and musculoskeletal system related new events seen in all patients

The hematopoietic system, musculoskeletal system and gastrointestinal system related events were all resolved after 6 months of initiation of ERT. Only one new event was recorded in one patient which was pain between 24 and 36 months. Hematopoietic system and musculoskeletal system events were shown in Fig. 3.

Growth, puberty, and routine daily activities-related events decreased and remained stable in every 6 months after initiation of ERT.

## Discussion

In this study, the quality of life-reducing events was shown to be decreased after enzyme replacement therapy in patients with non-neuronopathic Gaucher Disease. Our study is the first one that aimed to evaluate the number of real-life based and GD related events in patients with GD before and after ERT. The treatment goals of ERT up to now focused on measurable treatment targets such as Hb and platelet values, liver and spleen volumes. However, even if these measurable treatment goals show statistically significant benefits, the effect on real-world daily living is unclear. To date, clinical events are investigated for other inborn errors such as Fabry Disease and in a randomized trial, slower progression of events was demonstrated in patients receiving ERT than the placebo group, indicating impact of treatment in their lives [11]. 

In the early 1990s first enzyme replacement therapy had FDA approval, then with several new recombinant enzyme molecules, ERT became first line therapy in GD. Primary end points of ERT are decrease in volumes of liver and spleen, also increase in hemoglobin and platelet levels. It has been demonstrated that increase in platelet levels, 1,5-fold within the first year after ERT, prevent bleeding and obviate the need for prophylactic splenectomy [12]. For 10 of 15 GD patients bleeding tended to be not only a problem that interrupted daily activities, but also a life-threatening problem. None of the patients had bleeding problems after one year of initiation of ERT, except one patient who still have abnormal menstrual bleeding despite treatment. It is known that bleeding problems are not only related to patients’ platelet count but also platelet dysfunction and clotting abnormalities play role in the pathogenesis of bleeding. It is been suggested that liver disease or enlarged spleen may induce rapid clearance of clotting factors and that ERT ameliorate both platelet functions and factor levels in GD patients [13]. Event-based outcomes also support the efficacy of treatment on bleeding problems in GD patients. Parallel to the improvement in bleeding problems, none of patients needed any transfusion after treatment.

Therapeutic goal of ERT for organomegaly is the decrease in liver and spleen volumes. However normal spleen or liver volume may not be expected in patients with massive splenomegaly despite treatment. In addition, some patients may have experienced with minimal increase in spleen volume due to nodules or post infarction fibrotic scars [14, 15]. The enlargement of the liver and especially of the spleen causes discomfort and reduced effort capacity for daily activities due to severe abdominal distension. Two of our patients had suffered severe abdominal distension before the treatment leading feeding difficulties. Those patients didn’t suffer vomiting, feeding difficulties or umbilical hernia after treatment but abdominal distension persisted after three years of ERT and also in long-term because of their massive splenomegaly. Although ERT may have inadequate effect on massive organomegaly in some patients, reduction of related events was observed.

Various skeletal pathologies are seen in GD, as bone deformities, osteopenia, osteoporosis, osteonecrosis, fractures, bone crises and severe bone pain requiring analgesics [16]. Preventing osteonecrosis, joint collapse and improvement of bone mineral density are the therapeutic goals for skeletal pathologies. In our study only one patient had vertebral fracture before treatment. Even though ERT has known effects on bone crises, infarctions and fractures, it has been recommended to monitor bone mineral density and to treat with calcium, vitamin D supplementation or medications as needed [17, 18]. All our patients were routinely investigated for bone mineral density and treated accordingly if it was abnormal. Functional disability developed in one patient (patient 2) due to severe scoliosis.

Pain also one of the major problems that impaired daily quality of life and %66 of patients had complaint of bone pain. After treatment it is expecting bone pain to resolve in %50 patients within one year [6, 19]. In our study patient two was suffering bone pain requiring analgesics at least one time in a month, impairing his quality of life. After ERT bone pain lessened that he didn’t need to have analgesics. Patient 13, wasn’t able to participate weekly sports in his school. Pain resolved after six months after ERT and he could perform all physical activities. Since pain has an important role on daily quality of life, it can be suggested that ERT can prevent events and enable daily routines to be continued without need for medications, even in cases where pain is not completely relieved. One patient after several years of treatment she had pain but she had additionally nephrocalcinosis and she was diagnosed rheumatoid arthritis. That is why we were not able to distingue the cause of pain and understand that pain is associated with GD.

Pulmonary involvement is seen only 1–2% of patients with Type 1 GD and characterized by interstitial lung disease or pulmonary vascular disease like pulmonary hypertension or hepatopulmonary syndrome [20]. Some adjuvant therapies or high dose ERT may be required in some patients with respiratory system involvement [21]. None of our patients had any respiratory system related event. Besides one patient had to have inhaler therapy in long term follow up, after our study monitoring, due to interstitial lung disease. In a study with a 20 years follow-up period, pulmonary conditions such as interstitial/obstructive/restrictive lung disease, pulmonary hypertension, pulmonary hemorrhage, are shown to be a late and life treating complications. It is been suggested that incidence of lung disease is higher after 20 versus 10 years as GD patients treated with ERT [22]. Similarly, in terms of event-based outcomes, lung disease may be a long-term issue for GD patients.

Growth and puberty are two important parameter of monitoring Gaucher patients because it is known that impairment of growth and pubertal delay aren’t rare conditions for GD. In a study including 99 Pediatric Gaucher patients, height was found below five percentiles in %50 of Gaucher patients [23]. Also, it has been demonstrated that acceleration in growth can be achieved with ERT. Growth normalization was observed in 4 to 30 months in the same study, 36 months after treatment in another study [24]. Therapeutic goal of ERT for growth and puberty is to achieve a normal height for age within three years and normal onset of puberty. In our study, we have focused on growth retardation and pubertal delay necessitating pediatric endocrinology consultation and causing concern in the family. Only two of our six patients with growth retardation, achieved normal growth rate. Similarly, pubertal delay was seen approximately %50 of Gaucher patients. In our study pubertal delay was not that frequent. Increased energy requirement and relative malnutrition has been suggested to be the main cause of delay in growth and puberty, that could improve with ERT [25]. Although age and pubertal status of patients at the initiation of treatment may be determinative for growth in short term and also for the final height.

Performing daily physical activities and improving the quality of life are also important gains in patients with GD. Also, therapeutic goals of ERT include restoring physical function and reducing fatigue [10, 14]. Prior to their treatments, three of our patients were unable to work, and two of them were still unable to do so afterward, even in long term. Therefor ERT did not fully suffice in terms of events to improve physical function even if it was demonstrated that ERT improve quality of life of GD patients in previous studies [26]. More studies with long term follow-up period are needed to better elucidate those data.

The bleeding episodes, as events were found to be decreased after ERT in this study and this was consistent with the findings in the paper by Cohen et al., which showed that the risks of early PPH and red blood cell (RBC) transfusions were significantly lower when ERT was used during pregnancy [27].

In conclusion, this study evaluates GD related real-life based events in patients with GD before and after ERT. All patients were received ERT during their follow up period, thus the lack of the control group who were not receiving treatment was the limitation of our study. Over all real-life events decreased in number after initiation of ERT in all patients. On the other hand, it was noted that some patients continued to have physical incapacity or missed workdays despite ERT. This implies the importance of starting ERT before the “point-of-no-return” and also the importance of event-based evaluation.

## Electronic supplementary material

Below is the link to the electronic supplementary material.

Supplementary Material 1

## Data Availability

The data that supports findings of this study are not publicly available due to restrictions of the institution. However, data are available from authors upon reasonable request.

## Abbreviations

ERT: Enzyme replacement therapy
GCase: Glucocerebrosidase
GD: Gaucher disease
GlcCer: Glucosylceramide
GlcSph: Glucosylsphingosine
SRT: Substrate reduction therapy

## References

[1] Hruska KS, LaMarca ME, Scott CR, Sidransky E. Mutation and polymorphism spectrum in the glucocerebrosidase gene (GBA). Hum Mutat S. 2008;29:567–83. 10.1002/humu.20676 18338393

[2] Hughes D, Mikosch P, Belmatoug N, Carubbi F, Cox T, Goker-Alpan O, Kindmark A, Mistry P, Poll L, Weinreb N, Deegan P. Gaucher disease in bone: from pathophysiology to practice. J Bone Min Res. 2019;34(6):996–1013. 10.1002/jbmr.3734 PMC6852006 31233632

[3] Daykin EC, Ryan E, Sidransky E. Diagnosing neuronopathic gaucher disease: new considerations and challenges in assigning gaucher phenotypes. Mol Genet Metab. 2021;132(2):49–58. 10.1016/j.ymgme.2021.01.002 33483255 10.1016/j.ymgme.2021.01.002 PMC7884077

[4] Stirnemann J et al. The French Gaucher’s disease registry: clinical characteristics,complications and treatment of 562 patients. Orphanet J. Rare Dis., s. 2012;7:77. 10.1186/1750-1172-7-77 PMC3526516 23046562

[5] Stirnemann J, et al. A review of gaucher disease pathophysiology, clinical presentation and treatments. Int J Mol Sci. 2017;s 18:441. 28218669 10.3390/ijms18020441 PMC5343975

[6] Mistry PK, et al. Transformation in pretreatment manifestations of gaucher disease type 1 during two decades of Alglucerase/imiglucerase enzyme replacement therapy in the international collaborative gaucher group (ICGG) gaucher registry. Am J Hematol. 2017;929:929–39. 10.1002/ajh.24801 PMC5600096 28569047

[7] Weinreb NJ et al. Gaucher disease type 1 patients from the ICGG Gaucher Registry sustain initial clinical improvements during twenty years of imiglucerase treatment. 2021, Molecular Genetics and Metabolism, s. 132,100–111. 10.1016/j.ymgme.2020.12.295 33485799

[8] Baris HN, Cohen IJ, Mistry PK. Gaucher disease: the metabolic defect, pathophysiology, phenotypes and natural history. Pediatr Endocrinol Rev. 2014;12(Suppl 10 1):72–81. 25345088 PMC4520262

[9] Gupta P, Pastores G. Pharmacological treatment of pediatric gaucher disease. Expert Rev Clin Pharmacol. 2018;11(12):1183–94. 10.1080/17512433.2018.1549486 30444430 10.1080/17512433.2018.1549486

[10] Biegstraaten M, Cox TM, Belmatoug N, Berger MG, Collin-Histed T, Vom Dahl S, Di Rocco M, Fraga C, Giona F, Giraldo P, Hasanhodzic M, Hughes DA, Iversen PO, Kiewiet AI, Lukina E, Machaczka M, Marinakis T, Mengel E, Pastores GM, Plöckinger U, Rosenbaum H. S management goals for type 1 gaucher disease: an expert consensus document from the European working group on gaucher disease. Blood Cells, Molecules and Diseases. 2016. 10.1016/j.bcmd.2016.10.008 28274788

[11] Banikazemi M, Bultas J, Waldek S, Wilcox WR, Whitley CB, McDonald M, Finkel R, Packman S, Bichet DG, Warnock DG, Desnick RJ, Fabry Disease Clinical Trial Study Group. Agalsidase-beta therapy for advanced Fabry disease: a randomized trial. 2007, Ann Intern Med., s. Jan 16;146(2):77–86. 10.7326/0003-4819-146-2-200701160-00148 17179052

[12] Krasnewich D, Dietrich K, Bauer L, Ginns EI, Sidransky E, Hill S. Splenectomy in gaucher disease: new management dilemmas. Blood S. 1998;91:3085–7. 9531624

[13] Linari S, Castaman G. Hemostatic abnormalities in gaucher disease: mechanisms and clinical implications. J Clin Med S Nov. 2022;24(23):6920. 10.3390/jcm11236920 PMC9735904 36498496

[14] Pastores GM, Weinreb NJ, Aerts H, Andria G, Cox TM, Giralt M, Grabowski GA, Mistry PK, Tylki-Szymańska A. Therapeutic Goals in the Treatment of Gaucher Disease. 2004 Oct., Semin Hematol., s. 41;4–14. 10.1053/j.seminhematol.2004.07.009 15468045

[15] Weinreb NJ, Charrow J, Andersson HC, Kaplan P, Kolodny EH, Mistry P, Pastores G, Rosenbloom BE, Scott CR, Wappner RS. Zimran A.1028 patients with type 1 Gaucher disease after 2 to 5 years of treatment: a report from the Gaucher Registry. 2002, Am J Med, s. 113:112–119. 10.1016/s0002-9343(02)01150-6 12133749

[16] Wenstrup RJ, Roca-Espiau M, Weinreb NJ, Bembi B. Skeletal aspects of Gaucher disease: A review. 2002, J Radiol, s. 75:A2-A12. 10.1259/bjr.75.suppl_1.750002 12036828

[17] Hughes D, Mikosch P, Belmatoug N, Carubbi F, Cox T, Goker-Alpan O, Kindmark A, Mistry P, Poll L, Weinreb N, Deegan P. Gaucher Disease in Bone: From Pathophysiology to Practice. 2019, J Bone Miner Res., s. 2019;34(6):996–1013. 10.1002/jbmr.3734 PMC6852006 31233632

[18] Cappellini MD, Carubbi F, Di Rocco M, Giona F, Giuffrida G. Long-term bone outcomes in Italian patients with Gaucher disease type 1 or type 3 treated with imiglucerase: A sub-study from the International Collaborative Gaucher Group (ICGG) Gaucher Registry. 2023, Blood Cells Mol Dis, s. Jan;98:102705. 10.1016/j.bcmd.2022.102705 36327675

[19] Charrow J, Andersson HC, Kaplan P, Kolodny EH, Mistry P, Pastores G, Rosenbloom BE, Scott CR, Wappner RS, Weinreb NJ, Zimran A. The Gaucher registry: Demographics and disease characteristics of 1698 patients with Gaucher disease. 2000, Arch Intern Med, s. 160:2835–2843. 10.1001/archinte.160.18.2835 11025794

[20] Mistry PK, Sirrs S, Chan A, Pritzker MR, Duffy TP, Grace ME, Meeker DP, Goldman ME. Pulmonary hypertension in type 1 Gaucher’s disease: genetic and epigenetic determinants of phenotype and response to therapy. Mol Genet Metab S. 2002;77:91–8. 10.1016/s1096-7192(02)00122-1 12359135

[21] Pastores GM, Sibille AR, Grabowski GA. Enzyme therapy in Gaucher disease type 1: Dosage efficacy and adverse effects in 33 patients treated for 6 to 24 months. 1993, Blood, s. 82:408–4016. 8392397

[22] Huang YN, Huang JY, Liao WL, Chiang SL, Liu KW, Bau DT, Wang CH, Su PH. Incidence of Pulmonary and Respiratory Conditions in Gaucher Disease from 2000 to 2020: A Multi-institutional Cohort Study. 2023, In Vivo, s. Sep-Oct;37(5):2276–2283. 10.21873/invivo.13330 PMC10500493 37652520

[23] Kaplan P, Mazur A, Manor O, Charrow J, Esplin J, Gribble TJ, Wappner RS, Wisch JS, Weinreb NJ. Acceleration of retarded growth in children with Gaucher disease after treatment with alglucerase. 1996, J Pediatr, s. 129:149– 53. 10.1016/s0022-3476(96)70203-2 8757576

[24] Ida H, Rennert OM, Kobayashi M, Eto Y. Effects of enzyme replacement therapy in thirteen Japanese paediatric patients with Gaucher disease2001, Eur J Pediatr, s. 160:21–25. 10.1007/pl00008411 11195013

[25] Kauli R, Zaizov R, Lazar L, Pertzelan A, Laron Z, Galatzer A, Phillip M, Yaniv Y, Cohen IJ. Delayed growth and puberty in patients with Gaucher disease type 1: Natural history and effect of splenectomy and/or enzyme replacement therapy. 2000, Isr Med Assoc J, s. 2:158–163. 10804944

[26] Oliveira FL, Alegra T, Dornelles A, Krug BC, Netto CB, da Rocha NS, Picon PD, Schwartz IV. Quality of life of brazilian patients with Gaucher disease and fabry disease. 2013, JIMD Rep., s. 3;7:31– 7. 10.1007/8904_2012_136 PMC3573176 23430492

[27] Cohen Y, Frydman D, Rotem R, Kofman R, Zimran A, Revel-Vilk S, Grisaru-Granovsky S. Risk of postpartum hemorrhage in multiparous women with gaucher disease: A call for reconsidering enzyme replacement therapy in all pregnant patients. J Inherit Metab Dis. 2021;44(5):1165–117. 33829536 10.1002/jimd.12382

