# Supplementary material for: Event-based real-life outcomes of patients with non-neuronopathic Gaucher disease receiving ert
Source: Orphanet J Rare Dis. 2025 Jun 12;20:302. doi: 10.1186/s13023-025-03690-8 (PMC12160344; doi:10.1186/s13023-025-03690-8)
Supplement: Supplementary file 1 — Supplementary Material 1 [file 13023_2025_3690_MOESM1_ESM.docx]

Supplementary figures

NUMBER OF NEW EVENTS BEFORE AND AFTER INITIATION OF ERT

ERT

ERT

ERT

ERT

ERT

ERT

ERT

ERT

ERT

ERT

ERT

ERT

ERT

ERT

ERT

ERT

ERT

Supplementary Figures

CUMULATIVE NUMBER OF EVENTS BEFORE AND AFTER INITIATION OF ERT

ERT

ERT

ERT

ERT

ERT

ERT

ERT

ERT

ERT

ERT

ERT

ERT

ERT

ERT
